# Human TRPV1 Channels are Functional Allosteric Receptors for Ciguatoxins and Brevetoxins

**DOI:** 10.1021/acschemneuro.5c00833

**Published:** 2025-12-10

**Authors:** Uxía Rodríguez-Rodríguez, Carmen Vale, M. Carmen Louzao, Luis M. Botana

**Affiliations:** Department of Pharmacology, Pharmacy and Pharmaceutical Pharmacology. Veterinary School, University of Santiago de Compostela, Lugo 27002, Spain

**Keywords:** TRPV1, ciguatoxins, brevetoxins, allosteric, oxidative stress

## Abstract

Ciguatera poisoning (CP) is a foodborne illness caused by the consumption of seafood containing ciguatoxins (CTXs). There is a wide variety of symptoms associated with ciguatera poisoning; however, the origin and physiological cause of many of them remains still unclear. Although the primary effect of ciguatoxins and brevetoxins (BTX) is their effect in voltage-gated sodium channels, in this work, the effect of both toxins on human transient receptor potential vanilloid 1 (TRPV1) channels was investigated under different physiological conditions that may contribute to CP. The results obtained showed that different physiological conditions that may occur in the organism potentiated the effect of ciguatoxins on TRPV1. Among these conditions, low pH, the presence of oxidative stress products, or endogenous ligands increased the TRPV1 currents induced by CTX3C and hyperpolarized their activation voltage. In addition, neurotoxic shellfish poisoning symptomatology (NSP), caused by brevetoxins, was previously linked to TRPV1 channels; therefore, in this study brevetoxins and ciguatoxins were combined to evaluate their effects on TRPV1 channels. The results obtained demonstrated that brevetoxin 3 alone did not alter TRPV1 channel currents or their activation; however, in the presence of the endogenous ligand anandamide BTX3 effects were potentiated. Furthermore, an allosteric effect of ciguatoxins and brevetoxins was observed, since the simultaneous presence of 0.5 nM CTX3C with different concentrations of BTX activated TRPV1 channels, increasing their maximum current intensity and hyperpolarizing the activation voltage.

## Graphical abstract

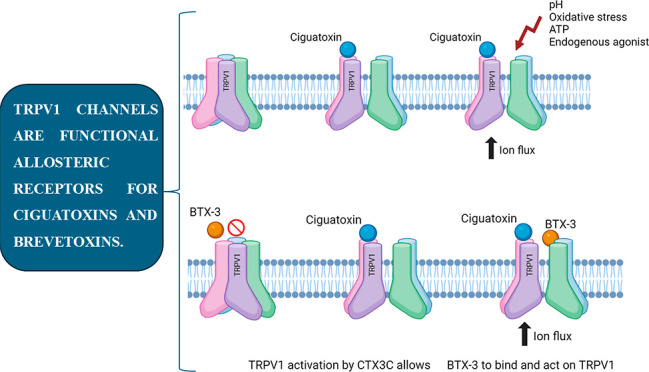

## Introduction

The consumption of seafood and fish containing ciguatoxins causes a worldwide illness named ciguatera poisoning. Ciguatoxins are produced by microalgae of the genera Gambierdiscus and Fukuyoa whose spatial and temporal distribution in European waters has increased over the last decades.^1^ The main risk of CP is the symptomatology produced, with over 175 known symptoms among which cardiovascular, neurological, and gastrointestinal alterations are the most common.^2^ Many of the symptoms of CTX poisoning are attributed to their effects on its main target, the voltage-gated sodium channels,^3–6^ however, other mechanisms are probably involved in CP symptomatology,^2^ and the origin of most of them is so far not understood. Tingling, prickling, and numbness in the lips, mouth, and face are frequently described in CP cases as well as cold allodynia.^2,7^ These sensory disturbances were previously associated with the interaction of CTXs with the transient receptor potential (TRP) channel family.^8^ All the members of this receptor family are ubiquitously expressed in the body and are considered nonselective cation channels involved in temperature and pain sensation.^9^ In particular, transient receptor potential vanilloid 1 could mediate some of the effects of CTX and BTX. TRPV1 receptors are expressed in multiple tissues such as epithelium, respiratory tract, gastrointestinal tract, urinary tract, and pancreatic and immune cells.^10^ These receptors are modulated by multiple endogenous and exogenous ligands (vanilloids, anandamide, proinflammatory mediators, ATP, toxins, and natural compounds) and physiological stimuli.^11^ Extracellular acidification or intracellular alkalinization^12^ and temperatures above 40 °C^13^ increase the TRPV1 channel opening. Oxidative stress products have also been reported as TRPV1 modulators.^14^ Different studies had demonstrated that CTXs act on TRPV1 receptors, being involved in some of the symptomatology presented in ciguatera including cold allodynia.^8,15^ Alterations in the perception of temperature have also been reported after exposition to brevetoxins,^16,17^ compounds causative of Neurotoxic Shellfish poisoning and produced by dinoflagellates of the genus *Karenia brevis*. However, in previous works BTXs did not cause direct effects over TRPV1 receptors.^18^

So far, it is assumed that BTXs and CTXs share their main cellular targets which are the voltage-gated sodium channels, but, in addition both groups of toxins have analogous chemical structure and their consumption through contaminated shellfish evokes similar symptomatology in humans.^1,2,19^ Indeed, the similarities between the physiological alterations observed after the consumption of BTXs and CTXs led previously to demonstrate their synergistic effect in human sodium channels.^20^ The worldwide spread of ciguatera poisoning^1,21–27^ and the scarce information about the mechanisms that trigger their long-lasting neurological symptomatology must be determined. In view of the different factors that modulate TRPV1 receptors, this work pursued to study the effects of these emergent toxins on TRPV1 under different pH conditions, oxidative stress products, intracellular ATP, and in the presence of the endogenous TRPV1 agonist anandamide. Additionally, the previous results demonstrating synergies between CTXs and BTXs in human sodium channels^20^ prompted the study of the combined effects of both toxins in TRPV1 receptors.

## Materials and Methods

### Chemicals and Toxins Used

Pacific ciguatoxin CTX3C was purchased from Wako (FUJIFILM Wako Chemicals Europe GmbH, Neuss, Germany) and dissolved in dimethyl sulfoxide (DMSO) at a final concentration of 1 μM. For experiments, subsequent solutions were made in Lockés buffer (154 mM NaCl, 5.6 mM KCl, 1.3 mM CaCl_2_, 1 mM MgCl_2_, 10 mM HEPES and 5.6 mM glucose, pH adjusted to 7.4 with Trizma base). Solvent solution was lower than 0.001% in all the experiments. Recordings of TRPV1 control currents were performed in the presence of the solvent alone under the same conditions. BTX-3 with 95% purity was purchased from Latoxan (France) and dissolved in DMSO at a final concentration of 50 μM. Consecutive dilutions were performed in Lockés buffer. DMSO concentrations during the experiments were lower than 0.001%. Capsazepine was purchased from Sigma and dissolved following manufacturer’s instructions in DMSO to use at a final concentration of 50 μM to antagonize TRPV1 currents as described for the HEK293 TRPV1 cell line.^28^ Consecutive dilutions were performed in Locke’s buffer, and the percentage of solvent used was lower than 0.01%. H_2_O_2_ (33% w/v) was purchased from Panreac (Spain) and consecutive dilutions were performed in Locke’s buffer. Acetic acid (CH_3_COOH) was used at a final concentration of 1 mM to decrease the extracellular pH up to 5.5. Anandamide was purchased from Merck and dissolved at a final concentration of 1 mM inDMSO and subsequent dilutions were made in Locke’s buffer to use at a final concentration of 1 μM. Also, in this case the percent of solvent was lower than 0.001%. All the currents were measured at the time point between 200 and 210 ms after the initiation of the voltage pulses.

### Human Cell Cultures

The human embryonic kidney cell line (HEK293) expressing the human TRPV1 channels were cultured in DMEM/F12 medium supplemented with 1% GlutaMAX, 1% nonessential amino acids solution (NEAA, Gibco), 10% fetal bovine serum, and 0.4 mg/mL Geneticin (G418, Gibco) and maintained at 37 °C in a humidified 95% O_2_/5% CO_2_ atmosphere, replacing the medium every 2 days. For electrophysiological experiments, cells were subcultured in 12-well plates in glass coverslips, coated with poly-d-lysine, at a density of 40,000 cel/mL.

### Electrophysiological Recordings

For whole cell patch-clamp recordings, cells were maintained at room temperature in a recording chamber with 0.5 mL Locke’s buffer as extracellular solution. The pH was maintained at 5.5 for experiments in acidic conditions or adjusted to 7.4 with a Trizma base for the rest of the electrophysiological experiments. Recording electrodes, fabricated with borosilicate glass microcapillaries (1.5 mm outer diameter), had resistances ranging from 5 to 10 MΩ. Two different pipet solutions were used to evaluate TRPV1 currents in the absence or in the presence of adenosine triphosphate (ATP). In normal conditions, the intracellular pipet solution contained (in mM): 120 mM CsF, 10 mM EGTA and 10 mM HEPES, and the pH was adjusted to 7.25 with Trizma base. The second intracellular solution contained (in mM): 120 NaCl, 10 MgCl_2_, 5 EGTA, 10 HEPES, and 2 mM Na_2_ATP (pH 7.25), since TRPV1 channels are highly sensitive to intracellular ATP.^29^ In all the experiments, cells were maintained at a holding potential (*V*_hold_) of −55 mV and 200 ms voltage steps from −100 to +100 mV in 10 mV increments were applied to record TRPV1 channel activation. In all the experiments, TRPV1 currents were measured every 5 min and the holding current did not change after bath application of compounds. Currents were obtained with a Multiclamp 700B amplifier and digitalized with the Digidata 1440A (both from Axon Instruments, California, U.S.A.). Signals were sampled at 50 kHz after low-pass Bessel filtering at 10 kHz and analyzed using pClamp 10 software (Axon Instruments). Series resistance was compensated by 70%.

### Statistical Analysis

All data are expressed as the means ± SEM of *n* determinations. Data analysis was performed using GraphPad Prism 8. Statistical comparisons were performed using one-way ANOVA followed by post hoc Dunnett’s test. *p* values <0.05 were considered statistically significant.

## Results

Symptomatology in CP, specifically the pathognomonic cold allodynia, was previously related to TRPV1.^18^ However, these channels are modulated by numerous stimuli and molecules that may alter the response of the channels to the toxin. Therefore, to understand the possible interaction of ciguatoxins with TRPV1, the currents through these channels in the presence of different toxin concentrations were analyzed under different conditions that may appear in the organism including extracellular acidification, endogenous ligands, oxidative stress, or an increase in intracellular ATP. Ciguatera and neurotoxic shellfish poisoning symptoms are very similar and include sensory disturbances, paraesthesia, and reversal of temperature perception.^8,15^ Taking into account that both groups of toxins may appear together in seafood products^1,16,17,22^ and the previously demonstrated synergism between ciguatoxins and brevetoxins in human sodium channels,^20^ the present work aimed to analyze the single and combined effects of pacific ciguatoxin CTX3C and BTX-3 in TRPV1 receptors using electrophysiological and pharmacological approaches.

### Effects of CTX3C in Human TPRV1 Channels

First, the effects of increasing CTX3C concentrations from 0.5 to 5 nM on TRPV1 channels after cell exposure to the toxins at physiological extracellular pH (7.4) were evaluated. Figure 1A,B represents the intensity–voltage relationship of TRPV1 currents and the representative recording traces at +100 mV. As shown in Figure 1A, CTX3C caused a concentration-dependent increase in the maximum current amplitude of TRPV1 currents, even at the lowest concentration evaluated. In control conditions, the maximum current at +100 mV was 1309 ± 100 pA (*n* = 15) increasing in a concentration-dependent manner up to 2552 ± 280 pA (*n* = 11; *p* < 0.0001) after the addition of 5 nM CTX3C. Representative traces of the currents elicited in control conditions and after bath-exposure of the cells to CTX3C are shown in Figure 1B. A bar graph summary of the effect of CTX3C over the maximum TRPV1 current amplitude is represented in Figure 1C, an effect that was ameliorated after bath addition of the TRPV1 antagonist capsazepine at 50 μM, which reduced the TRPV1 amplitude to 740 ± 196 pA (*n* = 7). Additionally, the activation voltage of TRPV1 was also evaluated and represented in Figure 1D. The activation voltage in control conditions was −14 ± 2.6 mV, −25 ± 2.3 mV in the presence of 0.5 nM CTX3C (*n* = 12; *p* = 0.014), −27.5 ± 3.5 mV after bath addition of 1 nM CTX3C (*n* = 9; *p* = 0.0087), and −27.7 ± 3 mV in the presence of 5 nM CTX3C (*n* = 11; *p* = 0.0075). In view of these results and since previous work^30^ have demonstrated that agonists such as capsaicin can desensitize TRPV1 receptors over time, the effect of the solvent over the different times of recording was evaluated and represented in Figure S1.

**Figure 1:**
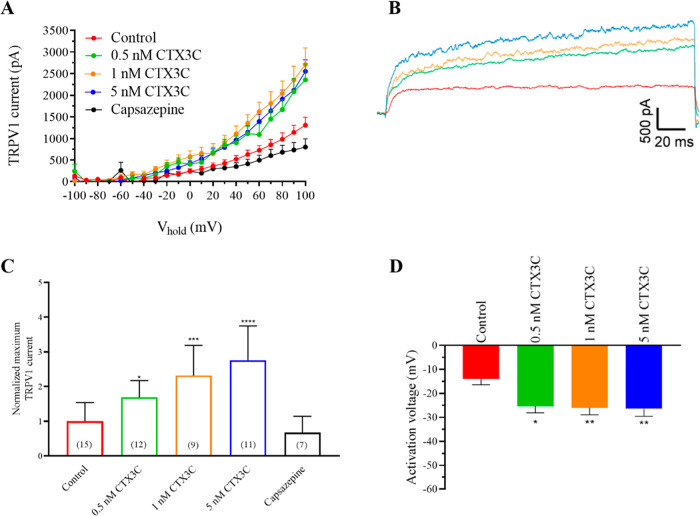
Effect of increasing CTX3C concentrations from 0.5 to 5 nM over TRPV1 channels. (A) Raw data of the intensity–voltage curves for the activation of TRPV1 channels in the absence and presence of CTX3C. (B) Representative recording traces of TRPV1 currents at +100 mV in control conditions (red trace) and after the addition of 0.5 nM (green), 1 nM (orange), and 5 nM (blue) CTX3C. (C) Summary of the maximum TRPV1 current intensity at +100 mV after the addition of CTX3C and the final addition of the antagonist capsazepine (cell numbers are indicated in parentheses). (D) Activation voltage in control conditions and after bath application of increasing CTX3C concentrations. *p < 0.05; **p < 0.01; ***p < 0.001; ****p < 0.0001 vs control. Data are expressed as mean ± SEM.

### Effects of CTX3C at Acidic pH in Human TPRV1 Channels

One of the conditions that are known to activate TRPV1 is acidic extracellular pH; thus, the effect of ciguatoxins on TRPV1 under these conditions (extracellular pH of 5.5) was studied. As represented in Figure 2A, the intensity–voltage curves show that the acidification of extracellular pH increased the activation of TRPV1 channels as previously described.^12^ In control conditions, the TRPV1 amplitude at +100 mV was 758.4 ± 146.9 pA (*n* = 9) at pH 7.4, 1651.3 ± 229.7 (*n* = 9) at pH 5.5, and further increased after bath application of 0.5 nM CTX3C to 2154.6 ± 234.1 pA (*n* = 8), to 3027.1 ± 366.9 pA (*n* = 8) in the presence of 1 nM CTX3C, and to 3585.4 ± 397.1 pA (*n* = 8) when 5 nM CTX3C was added to the bath solution. Figure 2B reflects the representative recording traces under each condition. Figure 2C represents the summary of the normalized current amplitude at +100 mV and their blockade after addition of 50 μM capsazepine at the end of each experiment. As represented in Figure 2D, the activation voltage in control conditions was −16.7 ± 2.9 mV at pH 7.4 (*n* = 9), −50 ± 5.5 mV (*n* = 9) at pH 5.5, −41.2 ± 6.1 in the presence of 0.5 nM CTX3C (*n* = 8), −55 ± 5.3 mV after bath addition of 1 nM CTX3C (*n* = 8), and −66.2 ± 4.6 mV in the presence of 5 nM CTX3C (*n* = 8). These results reveal the importance of pH in ciguatera intoxication, since lower pH may lead to higher TRPV1 maximum peak current intensity.

**Figure 2:**
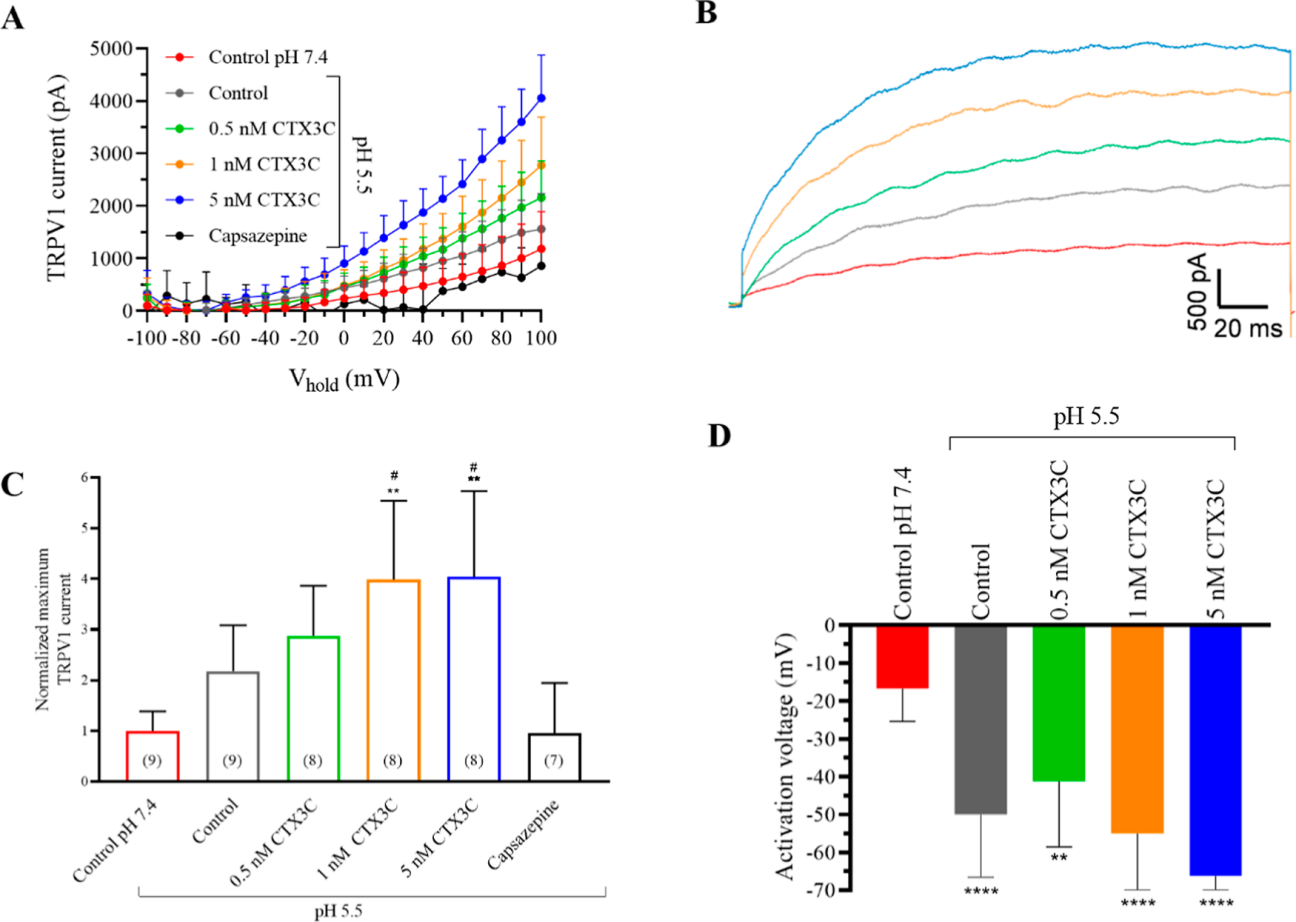
Effect of CTX3C on TRPV1 current amplitude in acidified extracellular medium. (A) Raw data of the effect of CTX3C in control conditions at pH 7.4, TRPV1 currents increased after the decrease of extracellular pH to 5.5 and further addition of increasing CTX3C concentrations from 0.5 nM to 5 nM. (B) Representative recording traces of TRPV1 currents at +100 mV in control conditions at pH 7.4 (red trace), control after the decrease of extracellular pH (gray trace), and after the addition of 0.5 nM (green), 1 nM (orange), and 5 nM (blue) CTX3C. (C) Normalized TRPV1 current intensity at +100 mV and after the addition of the antagonist capsazepine. (D) Activation voltage in control conditions at pH 7.4 in contrast to pH 5.5 and after bath application of increasing CTX3C concentrations. **p < 0.01; ****p < 0.0001 vs control conditions at pH 7.4. #*p* < 0.05 vs control conditions at pH 5.5. Data are expressed as mean ± SEM and the number of cells is represented in parentheses.

### Effects of CTX3C in Human TPRV1 Currents after Increasing Intracellular ATP

Since previous studies demonstrated that intracellular ATP regulates the activity of TRPV1,^29^ the effects of ciguatoxins on TRPV1 were analyzed by adding 2 mM ATP to the intracellular solution. In control conditions, TRPV1 currents reached 1120 ± 77 pA (*n* = 9). As represented in Figure 3A and the corresponding traces illustrated in Figure 3B, CTX3C increased TRPV1 currents to 4024.5 ± 430.2 pA (*n* = 9) after bath application of 5 nM CTX3C, an increase that was higher than that observed with intracellular solutions without ATP. Normalized data at +100 mV are represented in Figure 3C, in control conditions, after the application of 5 nM CTX3C and at the end of each experiment when 50 μM of capsazepine was added to block the TRPV1 currents as previously documented.^16^ Noteworthy, after capsazepine application, the TRPV1 current amplitude decreased to 521.5 ± 227.3 (*n* = 8). Furthermore, the activation voltage was hyperpolarized in the presence of CTX3C, being −6.4 ± 2 mV in control conditions and −29 ± 2.3 (*n* = 9; *p* = 0.0001), as represented in Figure 3D These results show that intracellular ATP increased the ciguatoxin effect on maximum TRPV1 current intensity and negatively shifted the activation voltage of TRPV1 channels, which is in accordance with previous data that documented this parameter to be affected by intracellular ATP.^29^

**Figure 3:**
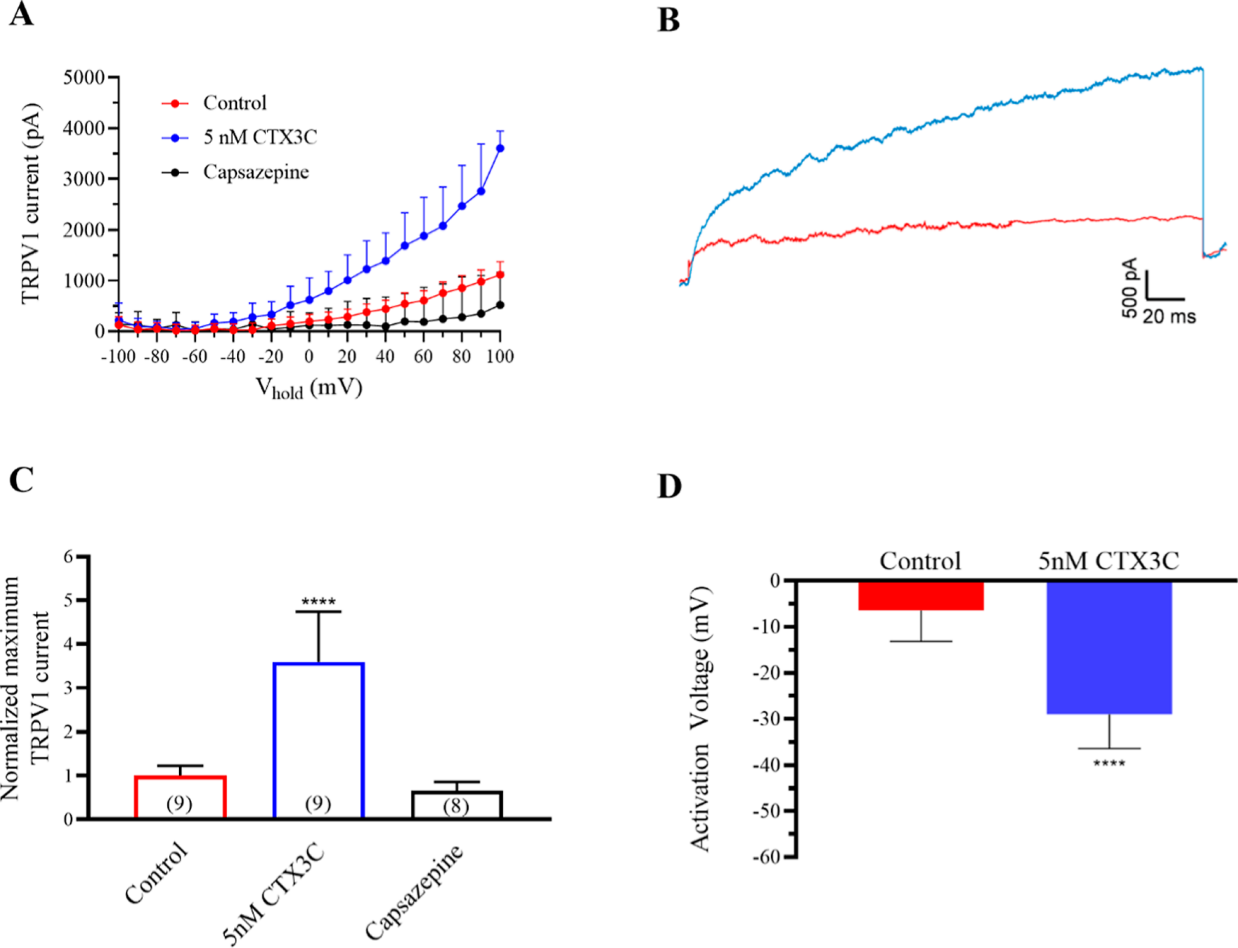
Effect of CTX3C on TRPV1 in the presence of 2 mM intracellular ATP. (A) Current–voltage relationship showing the average TRPV1 current intensity in control conditions and after bath application of 5 nM CTX3C and final addition of 50 μM capsazepine. (B) Representative recording traces at +100 mV in control conditions (red trace) and after bath application of 5 nM CTX3C (blue trace). (C) Normalized maximum TRPV1 current at +100 mV. (D) Activation voltage in control conditions and after bath application of 5 nM CTX3C concentrations. Data are expressed as mean ± SEM and the number of cells is presented in parentheses. ****p < 0.0001 vs control.

### Effects of CTX3C under Oxidative Stress Conditions in Human TPRV1 Channels

Oxidative stress conditions modulate TRPV1 channels.^14,31,32^ To evaluate the effects of ciguatoxins on TRPV1 channels under oxidative stress conditions, cells were exposed to hydrogen peroxide (H_2_O_2_), one of the reactive oxygen species (ROS) products. Initially, the effect of H_2_O_2_ alone was tested on TRPV1 channels at concentrations from 1 to 50 μM. As summarized in Figure 4A, according to previous reports, an increase of TRPV1 currents was recorded at concentrations of 50 μM H_2_O_2_,^14^ while none of the concentrations altered the activation voltage of the channels as represented in Figure 4B. At the end of each experiment, 50 μM capsazepine was also added to block TRPV1 currents.

**Figure 4:**
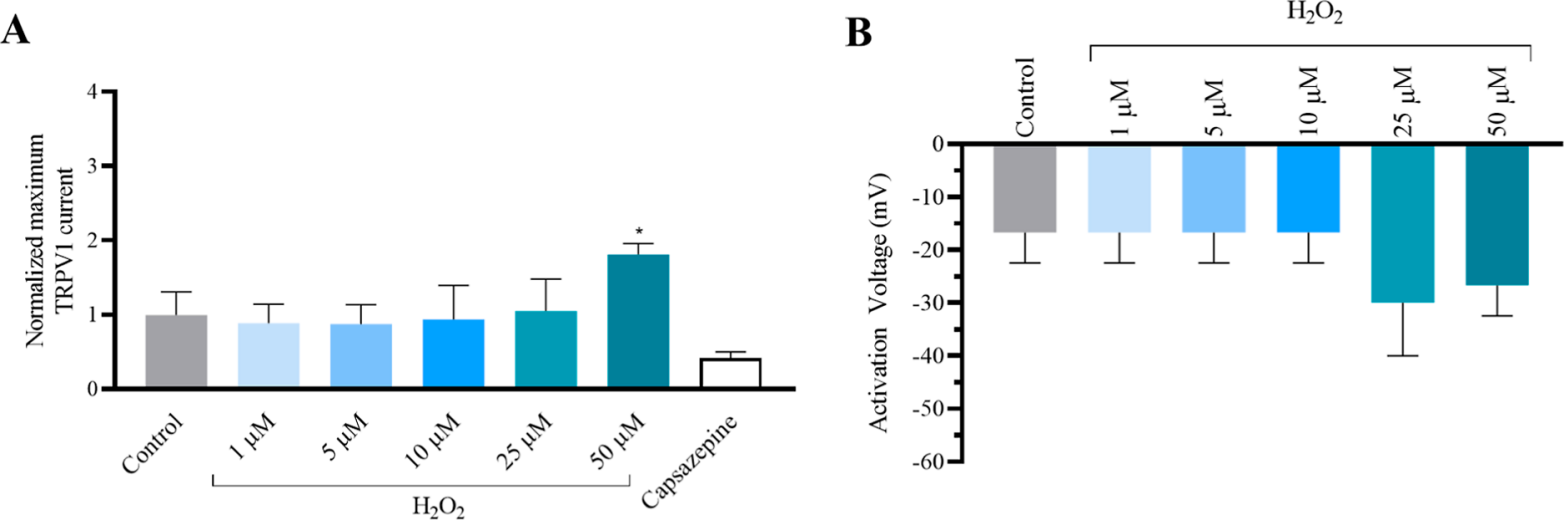
Effect of increasing H_2_O_2_ concentrations on TRPV1 current amplitude. (A) Averaged maximum TRPV1 current intensity at +100 mV normalized to the control. (B) Activation voltage in control conditions and after bath application of increasing H_2_O_2_ concentrations. **p < 0.05 vs control conditions.

Since a concentration of 5 μM·H_2_O_2_ is the highest concentration considered within the physiological range^33–36^ and did not affect TRPV1, the following experiments aimed to evaluate the effect of ciguatoxin in the presence of oxidative stress, using 5 μM H_2_O_2_. The results obtained are represented in the current voltage curves shown in Figure 5A and the corresponding representative traces at +100 mV in Figure 5B. The results indicate that 5 μM H_2_O_2_ in the bath did not affect maximum currents (1040.6 ± 72 pA in control conditions (*n* = 11) and 1472.4 ± 88 pA in the presence of 5 μM H_2_O_2_, (*n* = 10). Furthermore, CTX3C increased the TRPV1 currents in a dose-dependent manner. Thus, after bath addition of 0.5 nM CTX3C, TRPV1 currents were 2315.2 ± 183.1 pA (*n* = 10), 2797.4 ± 176.7 pA (*n* = 8) in the presence of 1 nM CTX3C, and further addition of 5 nM CTX3C induced an increase in TRPV1 currents up to 4143.2 ± 393 pA (*n* = 7) which is a remarkable higher increase than that observed only with the same concentrations of CTX3C. The decrease of the currents after the addition of capsazepine at the end of the experiment, as summarized in Figure 5C, confirmed that these effects were mediated by TRPV1 channels. The activation voltage of TRPV1 channels was also evaluated and represented in Figure 5D. The results showed that in the presence of 5 μM H_2_O_2_ the activation voltage of TRPV1 channels was shifted negatively at the lowest CTX3C concentration evaluated of 0.5 nM. Consequently, the negative shift in the TRPV1 activation voltage caused by CTX3C was higher in the presence of H_2_O_2_. Thus, the activation voltage of TRPV1 channels in control conditions was −13.6 ± 2.4, −25 ± 2.7 mV after exposure to H_2_O_2_, −31 ± 2.3 mV after bath application of 0.5 nM CTX3C, −37.5 ± 2.5 mV in the presence of 1 nM CTX3C, and −47.1 ± 4.2 mV after cell exposure to 5 nM CTX3C in the presence of 5 μM H_2_O_2_.

**Figure 5:**
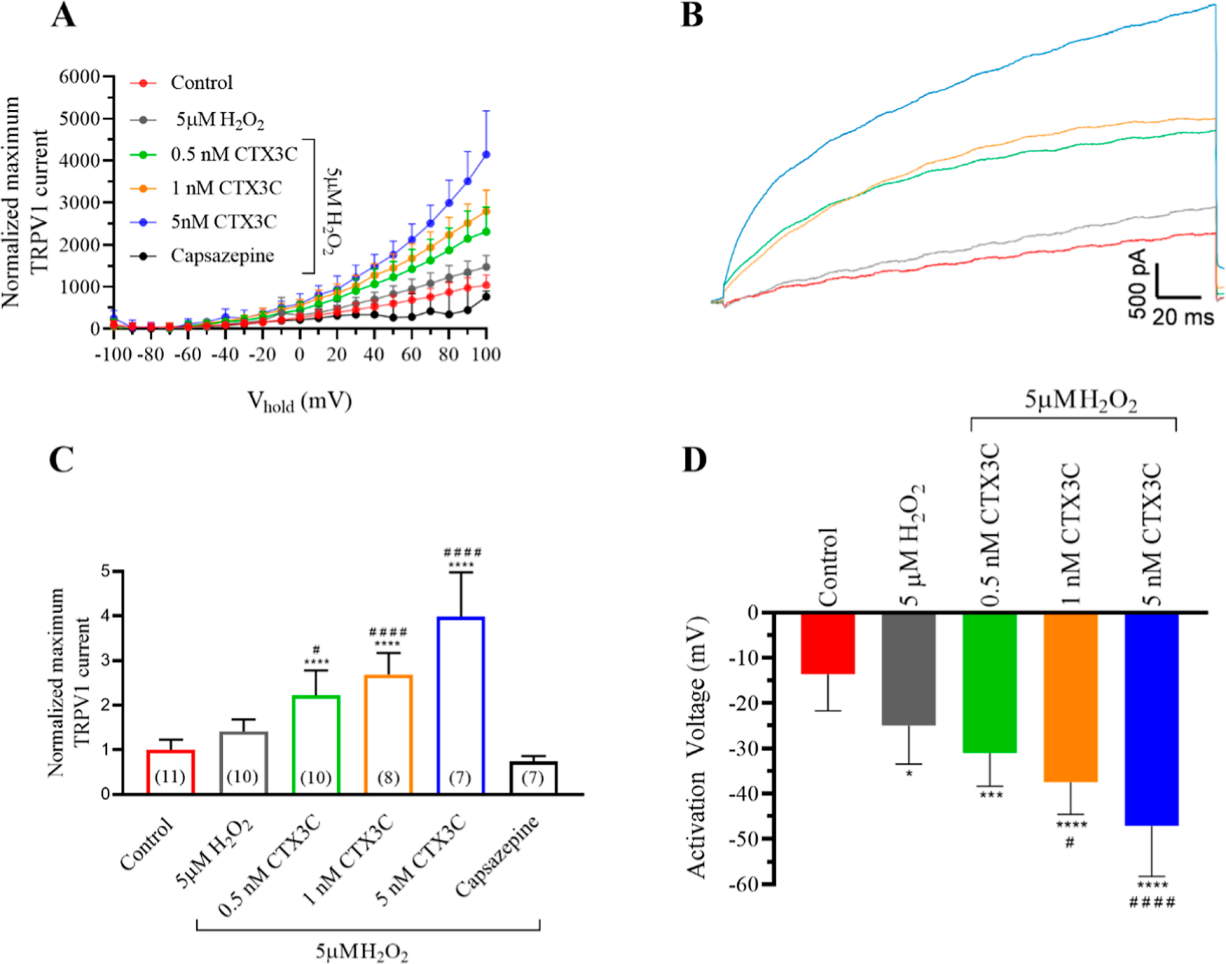
Effect of CTX3C on TRPV1 current amplitude in the presence of 5 μM H_2_O_2_. (A) Intensity–voltage curves for TRPV1 currents evoked by CTX3C in the presence of H_2_O_2_. (B) Representative recording traces of TRPV1 currents at +100 mV in control conditions (red trace), after the addition of 5 μM H_2_O_2_ (gray trace), and after additional addition of 0.5 nM (green trace), 1 nM (orange trace), and 5 nM (blue trace) CTX3C. (C) Summary of the maximum TRPV1 current intensity at +100 mV in the different conditions evaluated and after the addition of the antagonist capsazepine. (D) Activation voltage in control conditions and after bath application of increasing CTX3C in the presence of H_2_O_2_. Data are expressed as mean ± SEM. The number of cells is presented in parentheses. *p < 0.05; ***p < 0.001; ****p < 0.0001 vs control. #*p* < 0.05; ####*p* < 0.0001 vs 5 μM H_2_O_2_.

### Effects of CTX3C in the Presence of the Endogenous TPRV1 Agonist Anandamide

Anandamide is an endogenous ligand that has a lower efficacy for TRPV1 channels than capsaicin and significantly lower potency.^37^ Anandamide was reported to be active in the organism at concentrations between 0.7 to 10 μM.^37^ Figure 6A and the representative traces in Figure 6B illustrate that the presence of a low anandamide concentration of 1 μM caused higher maximum TRPV1 currents in the presence of 5 nM CTX3C than these registered for ciguatoxin alone. Cell exposure to anandamide increased TRPV1 currents from 1167.4 ± 64.6 pA (*n* = 11) to 1627.4 ± 92 pA (*n* = 10). Further addition to the bath of CTX3C led to a dose-dependent increase in current intensity, which was 1812.7 ± 70.4 pA (*n* = 10) in the presence of 0.5 nM CTX3C, 2226.1 ± 135.5 (*n* = 10) for 1 nM CTX3C, and 3173.5 ± 279.1 (*n* = 7) after bath application of 5 nM CTX3C. These data are summarized in Figure 6C with the TRPV1 antagonist capsazepine added at the end of each experiment. The activation voltage was also evaluated as represented in Figure 6D showing that while 1 μM anandamide did not affect this parameter, the addition of 1 nM CTX3C in the presence of anandamide shifted the activation voltage from −16 ± 3.4 mV (*n* = 10) in the cells treated with anandamide alone to −24 mV ± 2.2 pA (*n* = 10) in the presence of anandamide and 1 nM CTX3C. Further addition of 5 nM CTX3C caused a higher negative shift in the activation voltage of TRPV1 channels to −37.1 ± 2.9 (*n* = 7). These results are listed in Figure 6D.

**Figure 6:**
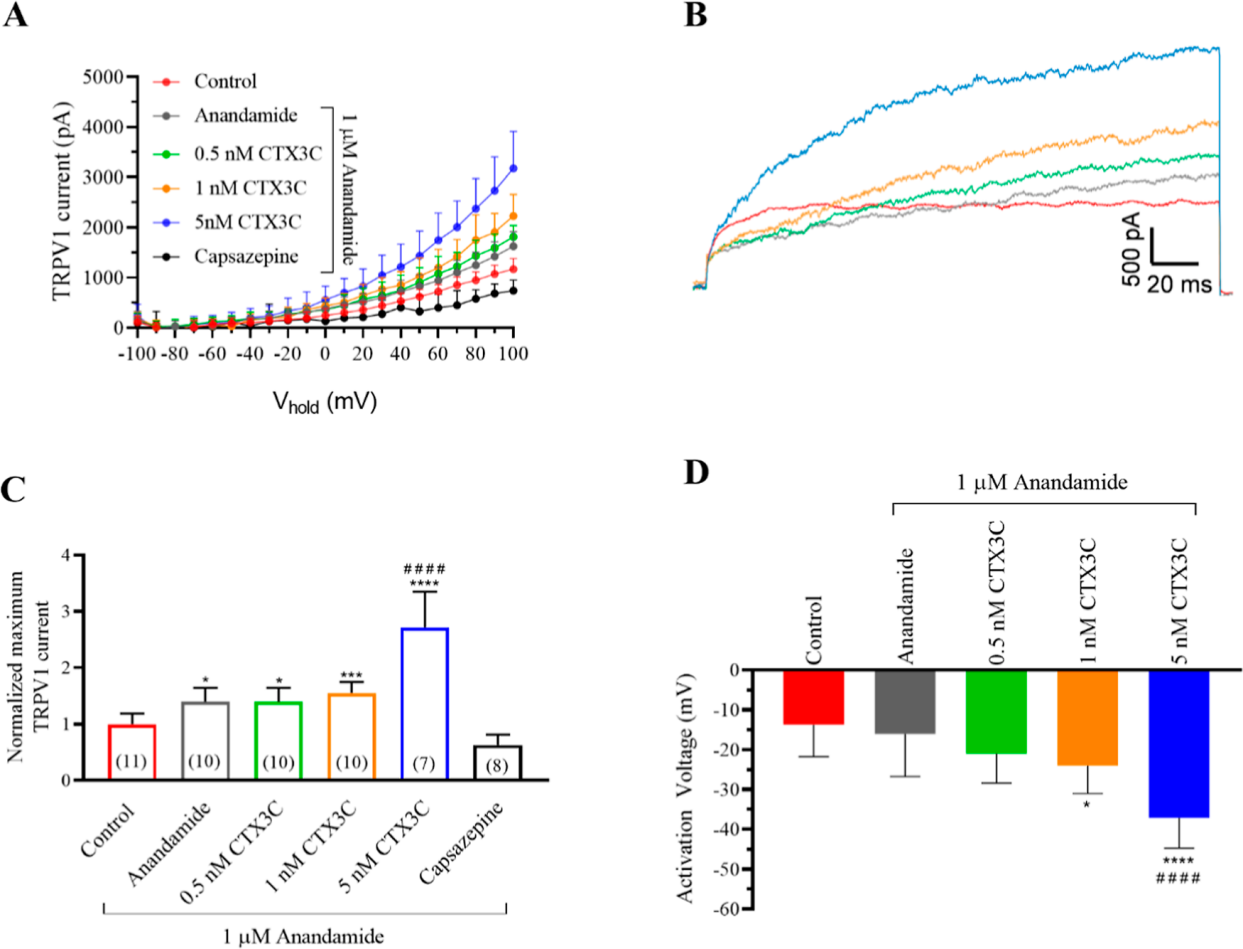
Effect of CTX3C on TRPV1 current amplitude in the presence of 1 μM anandamide. (A) Intensity–voltage relationship for TRPV1 currents activated by CTX3C in the presence of anandamide. (B) Representative recording traces of TRPV1 currents at +100 mV in control conditions (red trace), after the addition of 1 μM anandamide (gray trace), and after additionally adding 0.5 nM (green trace), 1 nM (orange trace), and 5 nM (blue trace) CTX3C. (C) Summary of the normalized maximum TRPV1 current intensity at +100 mV under the same conditions and after the addition of the antagonist capsazepine. (D) Activation voltage in control conditions and after bath application of anandamide and increasing CTX3C concentrations. Data are expressed as mean ± SEM and the number of cells is presented in parentheses. *p < 0.05; ***p < 0.001; ****p < 0.0001 vs control. ####*p* < 0.0001 vs 1 μM anandamide.

### Effects of BTX-3 Combined with CTX3C on Human TRPV1 Channels

The nervous symptoms triggered by NSP were previously related to alterations in temperature perception^17,38^ and this fact led us to study the BTX-3 effects on TRPV1 as it occurs in CP.^15,23^ Therefore, in the next set of experiments, the combined effects of ciguatoxins and brevetoxins in TRPV1 channels were evaluated using an extracellular pH of 7.4. In order to do this, first the effects of different BTX-3 concentrations on TRPV1 currents were analyzed. As represented in Figure 7, brevetoxins alone did not affect TRPV1 channels. Figure 7A represents the current–voltage curves under control conditions and in the presence of increasing BTX-3 concentrations (from 10 to 100 nM). The representative traces are shown in Figure 7B. Normalized TRPV1 currents at +100 mV are shown in Figure 7C showing that bath applications of 10, 50, or 100 nM BTX-3 did not affect TRPV1 maximum currents. At the end of each experiment, 50 μM capsazepine was added. Figure 7D represents the activation voltage of TRPV1 channels illustrating that none of the BTX-3 concentrations studied affected the TRPV1 activation voltage, which was −6.1 ± 1.8 mV (*n* = 13) in control conditions and −6.6 ± 2.3 mV (*n* = 9) at the highest BTX-3 concentration tested of 100 nM.

**Figure 7:**
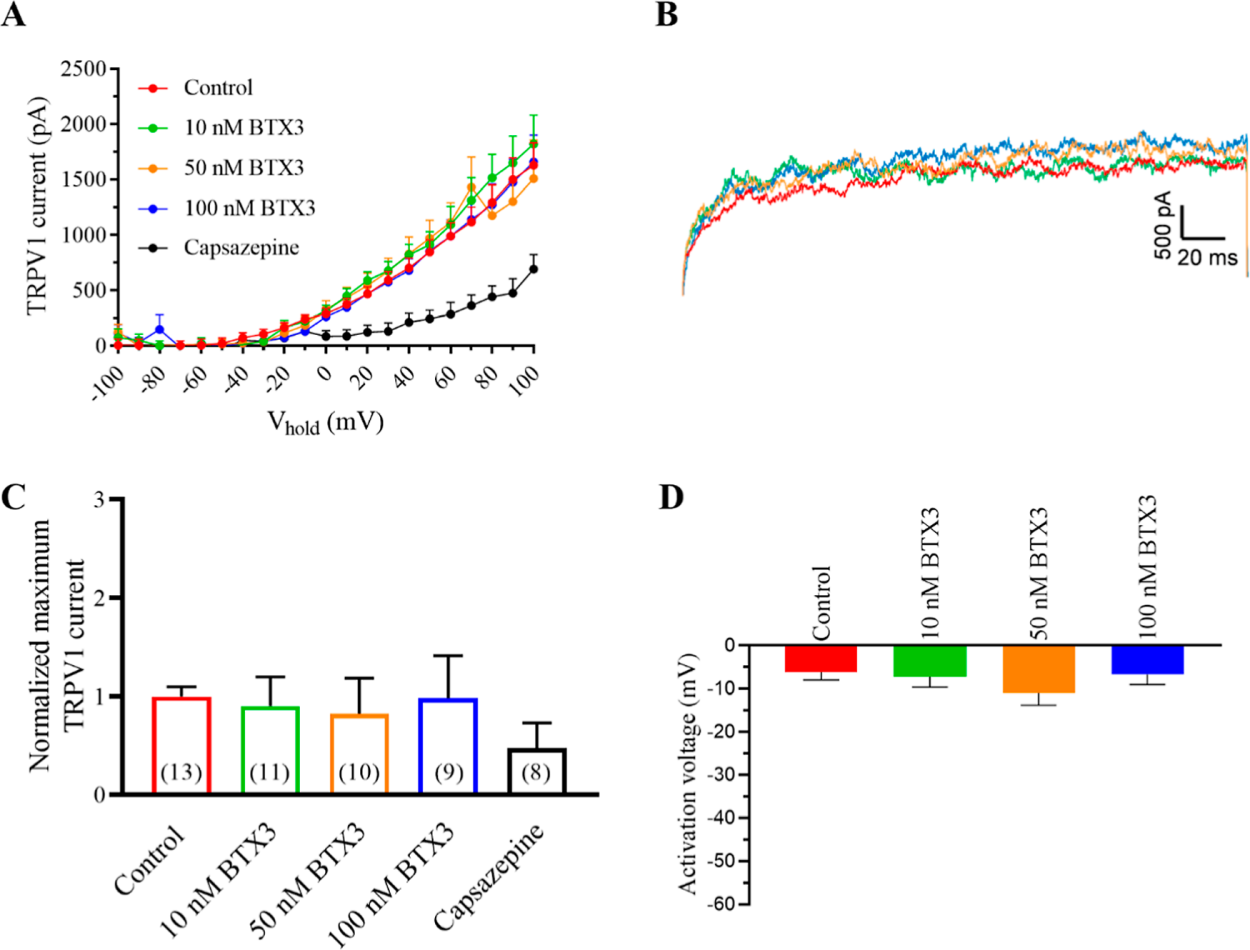
Effect of BTX-3 on TRPV1 channels. (A) Current–voltage relationship for the effect of different concentrations of BTX-3. (B) Representative traces of maximum TRPV1 current intensity in control conditions (red) and after bath addition of 10 nM (green), 50 nM (orange), and 100 nM (blue) BTX-3 at 100 mV. (C) Summary of the normalized maximum TRPV1 current intensity at +100 mV under the same conditions and after the addition of the antagonist capsazepine. (D) Activation voltage of TRPV1 channels in control conditions and after bath application of increasing BTX-3 concentrations. Data are expressed as mean ± SEM and the number of cells is presented in parentheses.

Since, previously, we have demonstrated a synergistic effect of CTXs and BTXs in human Na_v_1.6 voltage-gated sodium channels,^20^ in this study the combined effects of both marine biotoxins on TRPV1 currents were evaluated. As illustrated in Figure 8A (raw data for the intensity–voltage relationship) and 8B (representative traces at +100 mV), 0.5 nM CTX3C increased TRPV1 currents as previously described. However, the further addition of 10 nM BTX-3 significantly increased TRPV1 currents. In this case, in control conditions, maximum TRPV1 currents were 1400.7 ± 291 (*n* = 11), 2779.2 ± 270.7 (*n* = 11) in the presence of 0.5 nM CTX3C, and 3762 ± 441.8 mV (*n* = 10) after the addition of 10 nM BTX-3. Additionally, this increase in the currents elicited by BTX-3 was also statistically significant, in contrast to the increase elicited by CTX3C alone. These data are summarized in Figure 8C. This finding is important since, as previously described, cell exposure only to brevetoxin did not affect TRPV1.^18^ The study of the activation voltage of the channels is summarized in Figure 8D. While the addition of 0.5 nM CTX3C or 10 nM BTX-3 alone did not affect the activation voltage of TRPV1, when both compounds were added, the activation voltage was negatively shifted from −6 ± 2.2 mV (*n* = 11) in control conditions to −19 ± 2.3 mV (*n* = 10).

**Figure 8:**
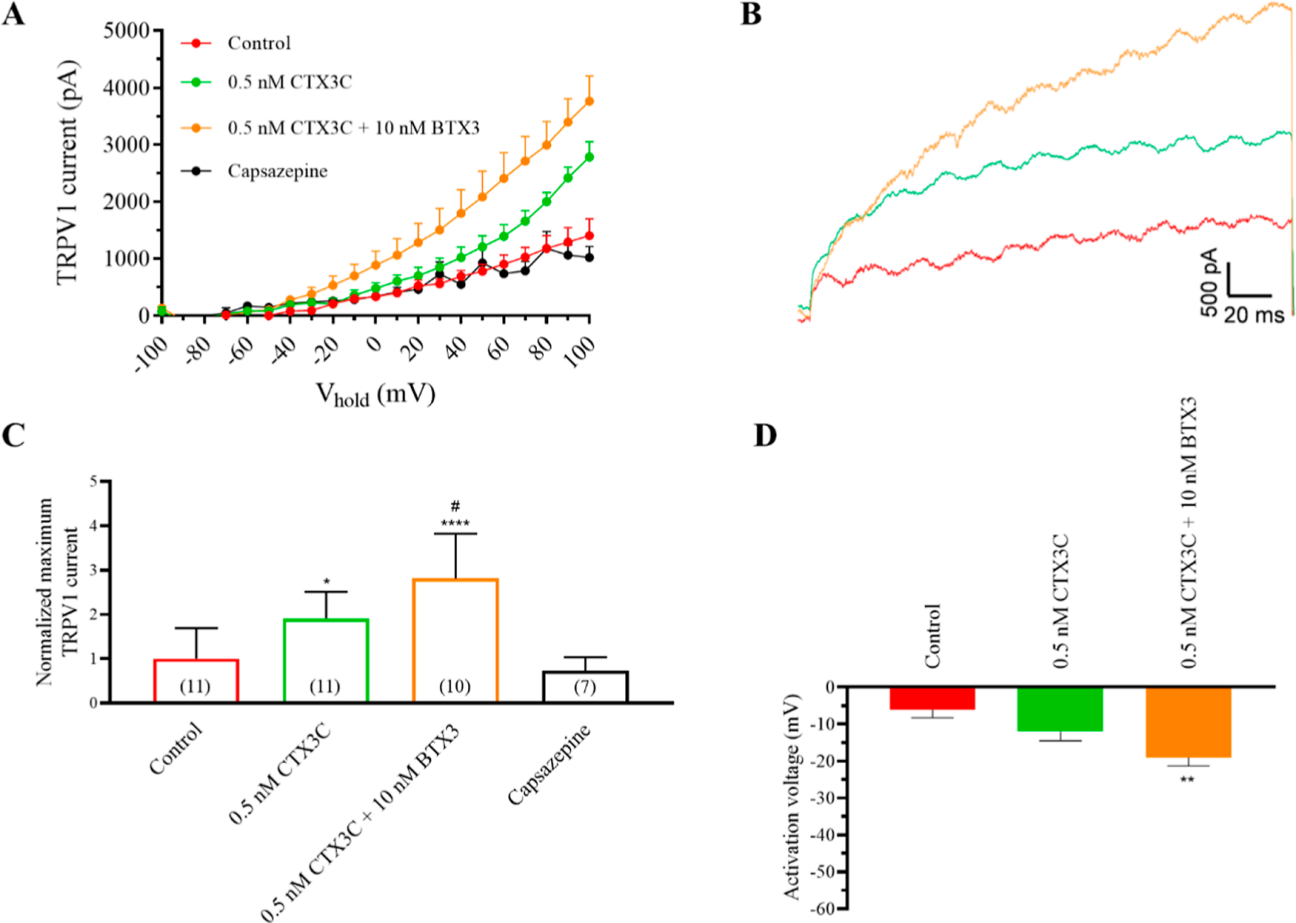
Effect of CTX3C and BTX-3 on TRPV1 channels. (A) Intensity–voltage curves representing the effect of CTX3C and their summatory effect with BTX-3 on TRPV1 currents. (B) Representative recording traces of TRPV1 currents at +100 mV in control conditions (red trace), after the addition of 1 μM anandamide (gray trace), and further addition of 0.5 nM (green trace), 1 nM (orange trace), and 5 nM (blue trace) CTX3C. (C) Summary of the maximum TRPV1 current intensity at +100 mV and after the addition of the antagonist capsazepine. (D) Activation voltage of TRPV1 channels in control conditions and after bath application of CTX3C and BTX-3. Data are expressed as mean ± SEM and the number of cells is presented in parentheses. *p < 0.05; **p < 0.01; ****p < 0.0001 vs control conditions. #*p* < 0.05 vs 0.5 nM CTX3C.

### Effects of CTX3C and TRPV1 Agonists in Human Naive HEK293 Cells

Finally, to confirm that the effects of CTX3C were specific for TRPV1 channels, capsaicin, anandamide, and CTX3C were evaluated in HEK293 cells not expressing TRPV1 channels. As shown in Figure 9, neither 1 μM anandamide nor 0.3 μM capsaicin or 5 nM CTX3C increased the outward currents present in naive HEK293. In these cells, the outward currents did not reach 1000 pA. These results are summarized in Figure 9A,B for anandamide, Figure 9C,D for capsaicin, and Figure 9E,F for 5 nM CTX3C, confirming that the results presented here are exclusively due to the effect of CTX3C on TRPV1 channels. In these cells, outward currents were 588.7 ± 29.2 (pA) in control conditions (Figure 9A) and 592 ± 41.5 (pA) after bath application of 1 μM anandamide. Normalized data at +100 mV are presented in Figure 9B. In that context, 0.3 μM capsaicin (Figure 9C,D) did not modify current amplitude which was 618.2 ± 43.5 (pA) in control conditions and 624.6 ± 51.6 (pA) in the presence of 0.3 μM capsaicin. Indeed, 5 nM CTX3C did not modify the amplitude of outward currents in cells not expressing TRPV1 channels which were 661.5 ± 32.6 (pA) in control conditions and 657.6 ± 26.7 (pA) in the presence of 5 nM CTX3C.

**Figure 9:**
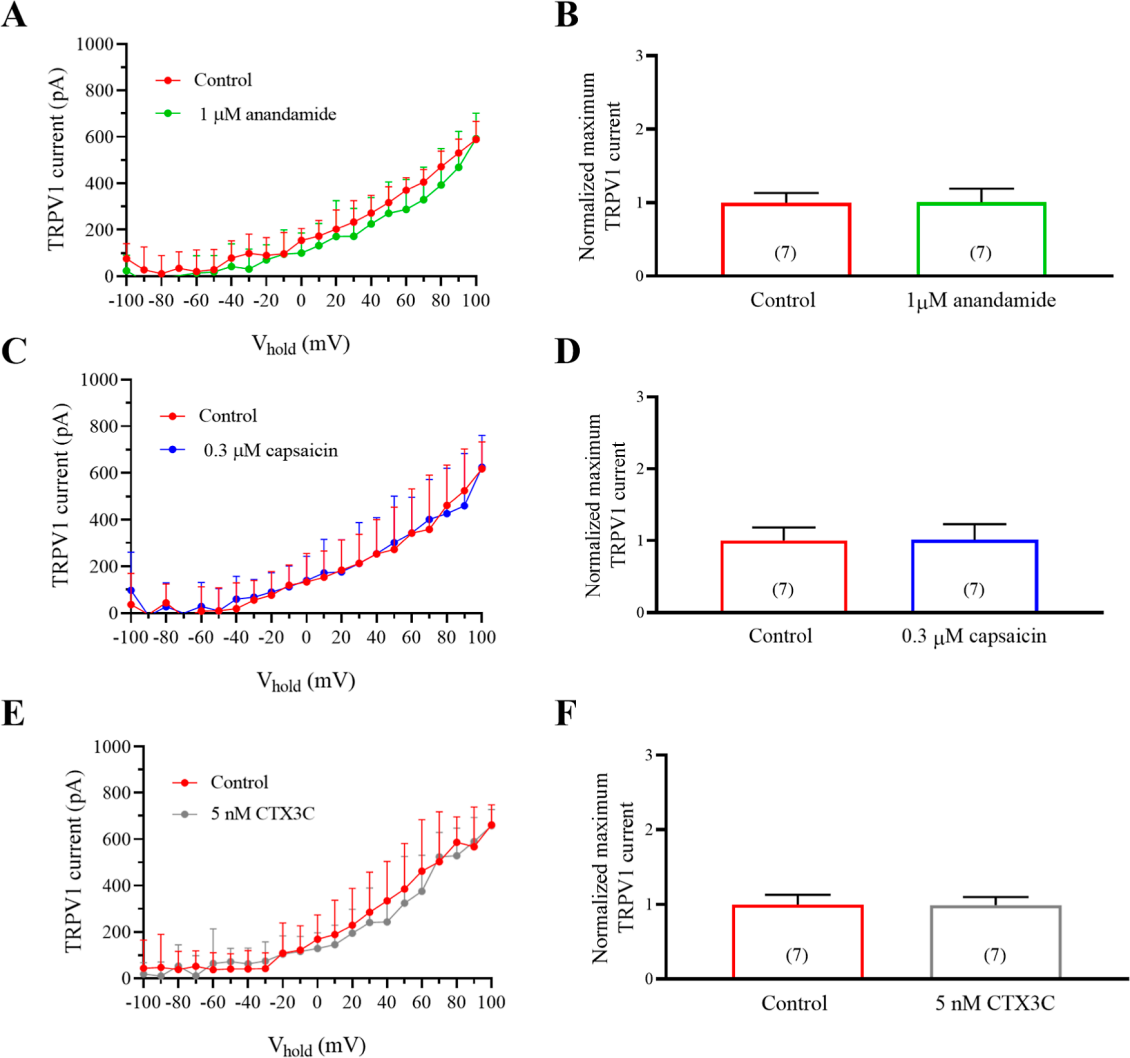
Effect of anandamide, capsaicin, and CTX3C in naive HEK293 cells without TRPV1 channels. Data are expressed as mean ± SEM and the number of cells is presented in parentheses.

## Discussion

The increasing expansion of marine biotoxins, specifically CP and the great variability of symptoms that appear in both the long and short terms, led to the development of this study with TRPV1 channels. Previous reports have related TRP channels to CP symptomatology^3,15,18^ given its involvement in temperature and burning-pain sensation^9,13,39^ and the important role of TRPV1 also in NSP.^18^ The implication of TRP channels in cold allodynia was explored in a previous work,^8^ but this study was focused on TRPA1 channels which are mainly located in dorsal root ganglion neurons and an absence of effect was described after whole-cell patch-clamp recordings.^8^ Since the response of these neurons was very sensitive to P-CTX-1 and greatly reduced by tetrodotoxin, leading to the conclusion that these symptoms were caused by the interaction of ciguatoxins with sodium and potassium channels combined with an effect on TRPA1 channels. This combined effect would allow the perception of burning pain and cold allodynia which are pathognomonic symptoms of CP. However, voltage increase does not fully activate the gating of TRP channels, and the allosteric interaction is proposed as the main activator of channel gating.^40^

Our results allow us to further expand the mechanism of cold allodynia caused by CP and describe for the first time the direct interaction of ciguatoxin with human TRPV1 channels. In this sense, it is noteworthy to remark that multiple recent studies have related TRPV1 with cold allodynia.^41–44^ These results are demonstrated by the allosteric interaction of CTX3C with anandamide and also with brevetoxins that are first reported in this study and supported by the fact that TRPV1 channels are a critical signal component of cold allodynia in sensory unmyelinated C-type afferent nerve fibers.^45^ In consequence, this mechanistic explanation, the direct interaction of ciguatoxins with TRPV1 channels, suggests that this receptor might be a candidate to explain one of the most puzzling effects of ciguatoxins. A second implication of this study is that ciguatoxins can no longer be considered only voltage-gated sodium and potassium channel modulators but also TRPV1-active toxins.

The fact that cold allodynia is a very relevant and persistent symptom caused by CP, suggests that the interaction with these channels could be an important component of their mode of action. Additionally, there are many factors that may alter TRPV1 functionality such as pH, intracellular ATP, temperature, or endogenous ligands,^45^ thus we studied how different conditions that can contribute to the symptoms observed after CP poisoning can affect the involvement of TRPV1 channels. Since TRPV1 channels are activated by acidic extracellular conditions,^12^ cell exposure to ciguatoxins in acidic extracellular pH enhanced the functional effects of ciguatoxins on TRPV1, increasing the amplitude of the currents and shifting the activation voltage of the channels to more negative potentials, closer to the resting potential of the cells. This fact is very important since TRPV1 channel hyperactivation is related to cold hyperalgesia and pain.^39,46^

Ciguatera crises and recurrence of the symptoms can be triggered by different factors such as sport activities, some foods, or alcohol consumption^26^ that can cause a decrease in pH and also the release of oxidative stress products. The liberation of oxidative stress products regularly occurs in the cells of the organism caused by an imbalance between production and accumulation of different reactive oxygen species as superoxide radicals, H_2_O_2_, and hydroxyl radicals.^47^ Hydrogen peroxide is produced by most cells in the human body. H_2_O_2_ concentrations vary in the intracellular fluid being 10 nM or lower^33,48^ while plasma fluid H_2_O_2_ concentrations are higher. These plasmatic concentrations measured under physiological conditions are between 0.25 and 5 μM,^33–36^ increasing after sport activities^49,50^ and reaching 50 μM in certain diseases or during inflammation.^33^ Previous reports have described a dose-dependent increase in TRPV1 currents at micromolar concentrations of H_2_O_2_.^14^ Based on the results obtained after the exposure of TRPV1 cells to different H_2_O_2_ concentrations, a conservative physiological H_2_O_2_ concentration of 5 μM was used. In the presence of oxidative stress products, such as H_2_O_2_, the increase in TRPV1 currents elicited by CTX3C was higher than that elicited by the same CTX3C concentration alone. All these findings are important when studying CP, when symptoms appear and when they are more pronounced. Thus, the data presented here suggest that under different physiological conditions in the organism, ciguatoxins may have different impact leading to a more pronounced symptomatology.

Moreover, intracellular ATP has been reported to regulate the basal activity of TRPV1 channels.^29^ In our study, the addition of ATP to the intracellular solution increased TRPV1 control currents without affecting the activation voltage of TRPV1 in the absence of ATP. However, an interesting finding was a greater negative shift in the activation voltage of the channels, which were activated at more negative membrane potentials in the presence of intracellular ATP and CTX3C.

Additionally, since the endogenous ligands for TRPV1 channels, is anandamide, a lipid mediator that acts as an agonist of TRPV1, with significantly lower functional efficacy than capsaicin,^37^ the effect of anandamide and CTX3C was evaluated. In this case, 1 μM anandamide, a concentration reported to be active in the organism,^37^ in the presence of CTX3C at concentrations ranging from 0.5 to 5 nM increased the effect of CTX3C, both in the current intensity and in the shift of the activation voltage of TRPV1 channels. Overall, these findings show that the effect of ciguatoxin is more pronounced under different conditions in the organism, which could explain the sudden appearance of crises and certain symptoms after consumption of ciguatoxins, even at very low concentrations.^3,15,23^

Along with CTX, BTX are marine biotoxins whose coexistence in fishery products has been previously reported.^16,51^ Both marine biotoxins act on sodium channels and have demonstrated synergistic effects on them.^20^ The intoxications derived from these toxins (CP and NSP) share some similar symptomatology; however, cell exposure only to BTX-3 did not affect TRPV1 channels, which is in accordance to previous data,^18^ a fact that suggests that the mechanism that triggers the symptoms seems to be different for brevetoxins and ciguatoxins. However, concomitant exposure of the cells to BTX-3 and low CTX3C concentrations led to an increase of 40% in TRPV1 currents. Additionally, the activation voltage of TRPV1 was also negatively shifted by BTX-3 when a low concentration of CTX3C was in the bath.

In summary, this study places an emphasis on the functional effects elicited by the simultaneous presence of ciguatoxins and brevetoxins in fishery products, demonstrating that there is an allosteric effect on the activation of TRPV1 channels, increasing the TRPV1 currents and shifting their activation voltage to the negative side when both BTXs and CTXs were combined. Brevetoxin is only able to bind and act on TRPV1 when the channel was previously activated by other molecule, as occurs with capsaicin^18^ or anandamide and in this work demonstrated that also with ciguatoxin. This fact becomes significant due to the important role of these channels in pain and temperature perception, and consequently, their involvement in CP and NSP.^9,18,52^ Therefore, the results presented here suggest that the long-lasting symptoms of CP and NSP could be ameliorated by the administration of TRPV1 channel antagonists.

## Supplementary Material



## Notes

Evaluation of the effect of the solvent over different times of recording (PDF)

## Abbreviations

ANOVA: analysis of variance
ATP: adenosine triphosphate
BTX: brevetoxins
CP: ciguatera poisoning
CTXs: ciguatoxins
P-CTX-1: pacific ciguatoxin-1
DMSO: dimethyl sulfoxide
EGTA: ethylene glycol-bis­(2-aminoethyl ether)-*N*,*N*,*N*′,*N*′-tetraacetic acid
HEK293: human embryonic kidney cell line
HEPES: 4-(2-hydroxyethyl)-1-piperazineethanesulfonic acid
NSP: neurotoxic shellfish poisoning
ROS: reactive oxygen species
SEM: standard error of the mean
TRP: transient receptor potential
TRPV1: receptor potential vanilloid 1
*V*_hold_: holding potential
w/v: weight volume ratio
