# Supplementary material for: Human TRPV1 Channels are Functional Allosteric Receptors for Ciguatoxins and Brevetoxins
Source: ACS Chem Neurosci. 2025 Dec 10;17(1):239–50. doi: 10.1021/acschemneuro.5c00833 (PMC12784398; doi:10.1021/acschemneuro.5c00833)
Supplement: Supplementary file 1 [file cn5c00833_si_001.pdf]

HUMAN TRPV1 CHANNELS ARE FUNCTIONAL ALLOSTERIC RECEPTORS FOR CIGUATOXINS AND BREVETOXINS

Uxía Rodríguez-Rodríguez, Carmen Vale, M. Carmen Louzao, Luis M. Botana**

Department of Pharmacology, Pharmacy and Pharmaceutical Pharmacology. Veterinary School, University of Santiago de Compostela, 27002, Lugo, Spain

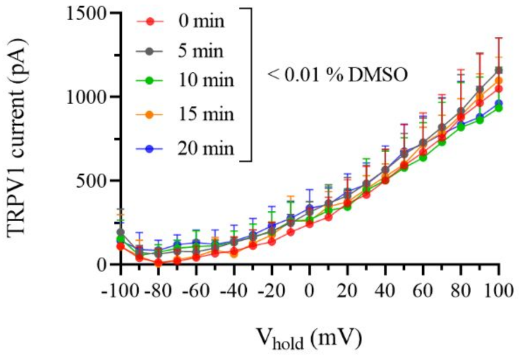

Supplementary Figure 1. TRPV1 receptors are not desensitized after solvent application over different times.
